# Predictive risk stratification model: a progressive cluster-randomised trial in chronic conditions management (PRISMATIC) research protocol

**DOI:** 10.1186/1745-6215-14-301

**Published:** 2013-09-18

**Authors:** Hayley A Hutchings, Bridie A Evans, Deborah Fitzsimmons, Jane Harrison, Martin Heaven, Peter Huxley, Mark-Rhys Kingston, Leo Lewis, Ceri Phillips, Alison Porter, Ian T Russell, Bernadette Sewell, Daniel Warm, Alan Watkins, Helen A Snooks

**Affiliations:** 1 Centre for Health Information Research and Evaluation (CHIRAL), College of Medicine, Swansea University, Singleton Park, Swansea SA2 8PP, UK; 2 Swansea Centre for Health Economics, College of Human and Health Sciences, Swansea University, Singleton Park, Swansea SA2 8PP, UK; 3 Abertawe Bro Morgannwg University Health Board, Morriston Hospital, Morriston, Swansea SA6 6NL, UK; 4 Mental Health Research Team, College of Medicine and College of Human and Health Sciences, Swansea University, Singleton Park, Swansea SA2 8PP, UK; 5 NHS Wales Informatics Services, 10-11 Old Field Road, Bocam Park, Pencoed CF35 5LJ, UK

**Keywords:** Predictive risk stratification, Clinical prediction models, Chronic disease, Primary care, Stepped wedge randomised trial

## Abstract

**Background:**

An ageing population increases demand on health and social care. New approaches are needed to shift care from hospital to community and general practice. A predictive risk stratification tool (Prism) has been developed for general practice that estimates risk of an emergency hospital admission in the following year. We present a protocol for the evaluation of Prism.

**Methods/Design:**

We will undertake a mixed methods progressive cluster-randomised trial. Practices begin as controls, delivering usual care without Prism. Practices will receive Prism and training randomly, and thereafter be able to use Prism with clinical and technical support. We will compare costs, processes of care, satisfaction and patient outcomes at baseline, 6 and 18 months, using routine data and postal questionnaires. We will assess technical performance by comparing predicted against actual emergency admissions. Focus groups and interviews will be undertaken to understand how Prism is perceived and adopted by practitioners and policy makers. We will model data using generalised linear models and survival analysis techniques to determine whether any differences exist between intervention and control groups. We will take account of covariates and explanatory factors. In the economic evaluation we will carry out a cost-effectiveness analysis to examine incremental cost per emergency admission to hospital avoided and will examine costs versus changes in primary and secondary outcomes in a cost-consequence analysis. We will also examine changes in quality of life of patients across the risk spectrum. We will record and transcribe focus groups and interviews and analyse them thematically. We have received full ethical and R&D approvals for the study and Information Governance Review Panel (IGRP) permission for the use of routine data. We will comply with the CONSORT guidelines and will disseminate the findings at national and international conferences and in peer-reviewed journals.

**Discussion:**

The proposed study will provide information on costs and effects of Prism; how it is used in practice, barriers and facilitators to its implementation; and its perceived value in supporting the management of patients with and at risk of developing chronic conditions.

**Trial registration:**

Controlled Clinical Trials ISRCTN no. ISRCTN55538212.

## Background

An ageing population and the associated increasing numbers of people with chronic conditions are placing unprecedented demands on health and social care services, both nationally and internationally [1-3]. New approaches to the management of chronic conditions are needed to shift the balance of care from the acute sector to primary and community sectors [4-6] through enhanced local services.

Clinical prediction models or risk scores are designed to predict a patient’s risk of having or developing a specified outcome or disease [7]. They use clinical findings (including medical history, drug use and test results) to make a diagnosis or predict an outcome [8]. As doctors either implicitly or explicitly use multiple predictors to assess a patient’s prognosis, multivariable approaches to the design of prediction models are more effective than single predictors [9]. Such prediction models are intended to help clinicians make better decisions by providing more objective estimates of probability as a supplement to other clinical information [9,10].

In 2008 the Wales Audit Office (WAO), UK, reported that NHS Wales was not providing services that fully supported the effective management of chronic conditions [11]. The report highlighted that 68% of admissions for chronic conditions were unplanned, and nearly 40% of admissions resulted in stays of less than 2 days. The new national policy for chronic conditions management in Wales is seeking to avoid the deterioration of existing chronic conditions by implementing a proactive, planned, integrated and generic approach to chronic conditions management across all sectors [6,12,13].

Three major research tasks have been identified that need to be completed before predictive risk tools can be routinely used in clinical practice: developing the prognostic model, validating its mathematical performance and evaluating its clinical performance [9,14-16]. The third task related to evaluating clinical performance is crucial, and the effect of a prognostic model on clinical behaviour and patient outcomes should be evaluated separately from the first two tasks [14]. While the number of prediction models is increasing, few have been validated [8] and evidence about their effects on patient care is limited. Reilly commented that, “without evaluation, clinicians cannot know whether using a prediction rule will be beneficial or harmful” [8]. Moons et al. suggested that formal validation and evaluation studies, ideally with random allocation of patients to intervention and control groups, can provide an opportunity to study factors that may affect the implementation of a prognostic model in daily care, including the acceptability and ease of use of the prognostic model to clinicians [14].

Although condition-specific risk prediction tools have been successfully developed and applied to conditions such as diabetes and coronary heart disease [17,18], there is less evidence regarding generic population-based tools. Predictive models, such as Patients at Risk of Readmission (PARR) and Scottish Patients At Risk of Readmission and Admission (SPARRA), have been used successfully in the UK National Health Service (NHS) to stratify patients into risk levels [19,20]. The models used in England and Scotland focussed only on those at most risk – on patients over 65 years in Scotland and on the sickest 1% or 2% in England. Steps to include the whole population were later included in the English Combined Predictive Model and are now being taken in Wales through the development of a predictive risk stratification model (Prism) [21]. Prism calculates a risk score of between 0 (no risk) and 100 (very high risk), based on patient demographics and data from primary and secondary care record systems. Patients are stratified into four levels based on their individual risk of having an emergency admission to hospital during the following year. This reflects the Welsh Chronic Conditions Management policy focus to prevent disease onset and deterioration across the population [6]. The performance of Prism appears comparable to or better than the English model [21] and an independent pilot evaluation [22] has indicated potential for impact. However, many practical questions remain about how it will be adopted and used by service providers for each risk stratum [23].

Although stratification will not in itself lead to improvements in service delivery, it aims to stimulate the planning and targeting of care. Thus it is intended to influence health care delivery and ultimately patient outcomes. Recent policy documents in the UK and internationally have generated expectations that, in future, health communities will routinely stratify their populations according to risk of hospital admission [1,2,6,24,25]. To inform future policy and practice we designed a prospective evaluation of the implementation of Prism and present the study protocol in this article.

### Study aim

To describe the processes of introducing a predictive risk stratification model (Prism) in Wales and to estimate its effects on the delivery of care, patient satisfaction, quality of life and resources used.

### Objectives

1) Measure changes in the profile of services delivered to patients across the spectrum of risk, focussing on emergency admissions to hospital.

2) Estimate the costs of implementing Prism and costs or savings associated with resulting changes in the utilisation of health and social care resources.

3) Assess the cost effectiveness of Prism by estimating cost per quality-adjusted life year based on changes in patient health outcomes.

4) Describe processes of change associated with Prism: how it is understood, communicated, adopted and used by practitioners, managers, local commissioners and policy makers.

5) Assess the effect of Prism on patient satisfaction.

6) Assess the technical performance of Prism.

### Design

We will undertake a mixed-methods progressive cluster-randomised trial with a quantitative evaluation sited within an area in southwest Wales and qualitative fieldwork across the whole of Wales. The main trial site, Abertawe Bro Morgannwg University Health Board (ABM UHB), is the second largest of seven health boards in Wales, serving around 600,000 people. It is divided into 11 GP practice clusters, within which there are 77 general practices. We will invite each of these practices to participate, with a target of 30–40 recruited practices.

The study fulfils the last of the three major steps (that of evaluating the clinical performance), in researching multivariable prognostic models identified by the recent series in the British Medical Journal [9].

**Figure 1:**
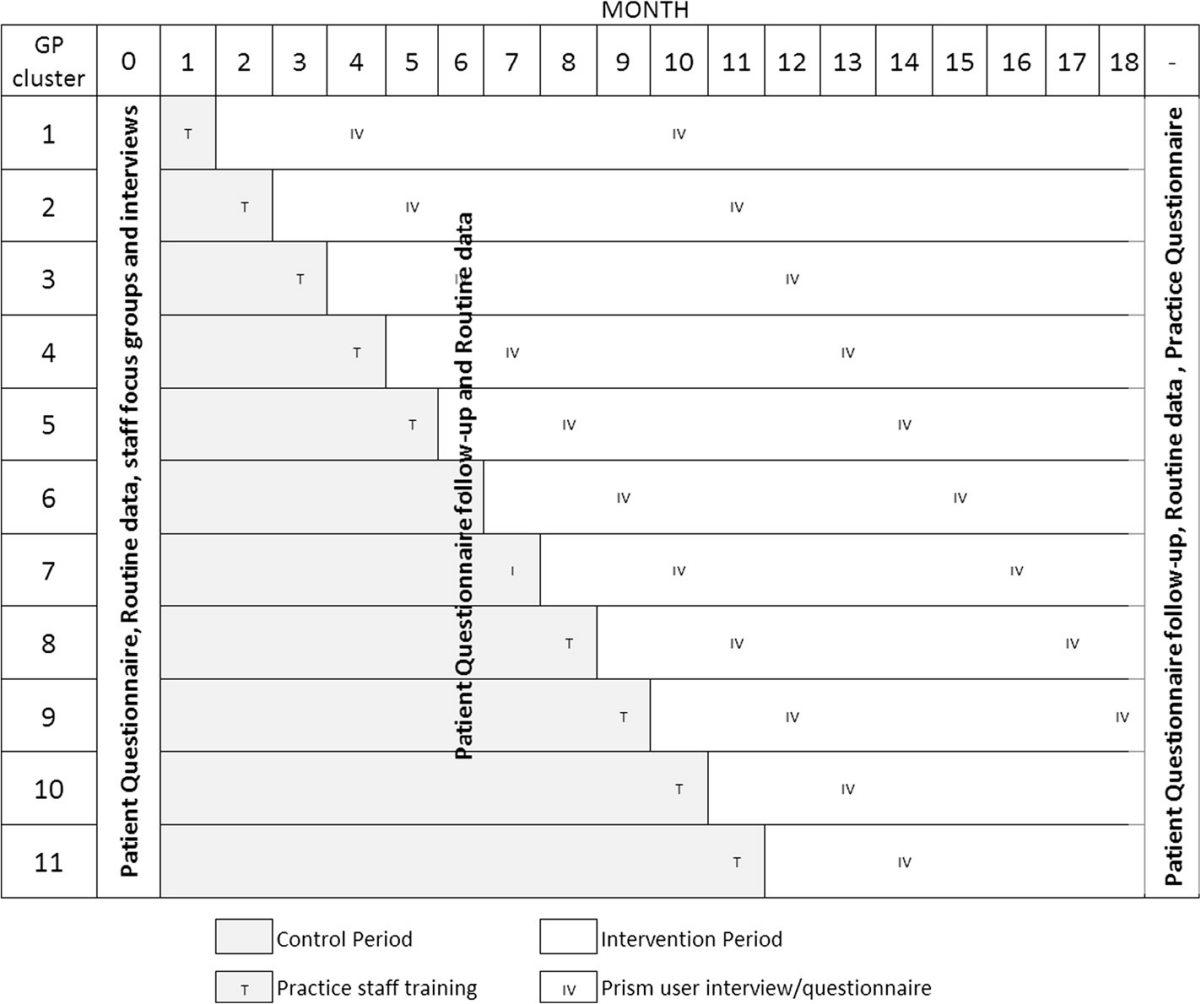
Randomised multiple interrupted time-series study design overview.

So that all participating practices have the opportunity to implement and use the Prism tool during the study period, we will use a progressive cluster-randomised trial design (randomised multiple interrupted time-series or stepped wedge design) [26-28] (see Figure 1).

All participating practices will begin as control practices without Prism; receive the Prism package and training; and thereafter be able to use Prism with clinical and technical support. Randomisation of practice clusters will be stratified by locality. The West Wales Organisation for Rigorous Trials in Health (WWORTH) will produce a random allocation schedule for the trial. Allocations will be concealed from the practices until 6 weeks prior to receiving the intervention. They will then be notified of the timescale for receipt of the intervention by telephone and email and training will be arranged before implementation of the intervention.

As the trial progresses, the number of intervention practices will increase and the number of control practices will fall. This design protects against many sources of bias, including inherent differences in study sites, contamination between practices, arbitrary changes in health policy and the ‘resentful demoralisation’ of controls deprived of the intervention.

### Prism

Prism is a web-based predictive risk tool commissioned by the Welsh Government that stratifies a General Practice population into four levels based on the individual risk of an emergency admission to hospital in the following 12 months. Prism was developed and checked using 300,000 (10% of the Welsh population) anonymised GP and hospital records from which 37 variables with the highest predictive power were selected. The variables used to develop Prism were drawn from routinely available data on inpatient, outpatient and primary care episodes and from the Welsh Index of Multiple Deprivation (http://wales.gov.uk/topics/statistics/theme/wimd/wimdstatement/;jsessionid=36B68DFE653760993E3E03966D7760B8?lang=en), which includes data on employment, income, housing, environment, education and health. To enable GP practices, individually or collectively, to plan workforce and resource allocation, each stratum represents a variable percentage of the practice population (which can be changed at the practice level depending on how they want to look at their own population data) with the top stratum (level 4) of patients being at highest risk of an emergency admission in the following year. The theoretical basis of the model is that patients in each of the four strata need very different targeted resources: level 4 requires individual case management, level 3 requires disease management on a population basis, level 2 requires supported self-care and level 1 needs prevention of illness and promotion of health and wellbeing.

### Intervention

**Figure 2:**
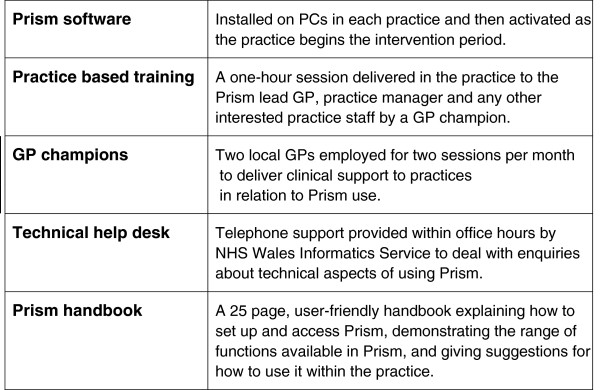
Components of the intervention.

The intervention comprises: Prism software, practice-based training; clinical support through two locally appointed ‘GP champions’, a telephone ‘help desk’ during working hours and a user-friendly handbook of guidance on using Prism including links to available Community Resource Teams that work at the locality level to provide multidisciplinary health and social care approaches to the assessment and management of more complex cases within the ageing community [29] (see Figure 2).

### Outcomes

Following Prism activation, we will compare between intervention and control groups:

#### Primary outcome

Number of emergency admissions per patient to hospital and time to first admission.

#### Secondary outcomes

Primary care service use—GP practice events/event days.

Accident and emergency attendances.

Community care service use.

Secondary care inpatient and outpatient episodes (including length of stays).

NHS implementation costs.

Number of Prism users.

Pattern (including frequency) of Prism use.

Patient satisfaction.

Predicted emergency admissions.

Health-related quality of life (SF-12).

We will also explore in detail within the intervention group and at other sites:

• Technical performance of the Prism tool—predicted compared to actual emergency admissions to hospital.

• Practitioner, commissioner and policy maker views about Prism implementation, adoption and effects.

## Methods/Design

To meet study objectives we will use anonymised linked routine data relating to processes of care for all patients registered at participating practices and will send postal questionnaires to a sample of patients at random, weighted to ensure inclusion of patients at the higher levels of risk. In addition, we will carry out focus groups and one-to-one interviews with service providers, commissioners, managers and policy makers. Figure 1 illustrates the continuous process of the trial and illustrates at what points during the trial we will collect data using the defined methods.

### Data collection and sources

Table 1 outlines the various data sources that will be collected, at what time points within the study these will be collected and how the data sources will used to answer our study objectives.

**Table 1:** Overview of methods employed in the study, matched to study objectives

**Objective**	**Data source**	**Sample**	**Collection time**
1. Measure changes in the profile of services delivered to patients across the spectrum of risk, focussing on emergency admissions to hospital	Anonymised routine linked data (including Prism data)	All patients from participating practices	Baseline
			6 months
			18 months
	Questionnaire data: Client Services Receipt Inventory (CSRI)	Random sample of patients from participating practices (*n* = 800 at each time point)	Baseline
			6 months
			18 months
2. Estimate the costs of implementing Prism and costs of resulting changes in the utilisation of health and social care resources	Questionnaire data: Client Services Receipt Inventory (CSRI); SF12	Random sample of patients from participating practices (*n* = 800 at each time point)	Baseline
			6 months
			18 months
	Structured telephone interviews	Prism users from all participating practices (*n* = up to 40)	18 months
3. Assess the cost effectiveness of Prism by estimating cost per quality-adjusted life year based on changes in patient health outcomes	Questionnaire data: SF12	Random sample of patients from participating practices (*n* = 800 at each time point)	Baseline
			6 months
			18 months
	Structured telephone interviews	Prism users from all participating practices	18 months
4. Describe processes of change associated with Prism: how it is understood, communicated, adopted and used by practitioners, managers, local commissioners and policy makers	Focus groups	GPs, practice nurses and managers from participating practices (*n* = 4); local health services managers and community staff managers (*n* = 1)	Baseline
	Interviews	GPs from participating practices who are unable to attend FGs (*n* = 12);	Baseline
		health board managers from sites not participating in main study (*n* = 6); policy makers and national health service managers (*n* = 5)	
	Interviews	Prism users from half of all participating practices, purposively sampled	3 months and 9 months after going live
	Questionnaire	Prism users from remaining half of all participating practices	3 months and 9 months after going live
	Focus group	Local health services managers and community staff managers (*n* = 1)	18 months
	Interviews	Health service managers from ABMU (*n* = 3)	18 months
	Structured telephone interviews	Prism users from all participating practices (*n* = up to 40)	18 months
5. Assess the effect of Prism on patient satisfaction	Questionnaire data: Quality of Care Monitor	Random sample of patients from participating practices (*n* = 800 at each time point)	Baseline
			6 months
			18 months
6. Assess the technical performance of Prism	Prism data	Prism risk data for patients at participating practices	Baseline
			6 months
			18 months
	Anonymised routine linked data	Routine health data	Baseline
			6 months
			18 months
	Structured telephone interviews	Prism users from all participating practices (up to 40)	18 months

#### Anonymised linked data

We will use routine data from the Secure Anonymised Information Linkage (SAIL) databank [30] to compare services delivered to patients (emergency, acute, primary, community and social care) across the spectrum of risk between intervention and control practices. SAIL includes routine Welsh hospital data such as emergency admissions (Emergency Department Data Set; EDDS), secondary care (Patient Episode Database for Wales, PEDW) and GP practice data. We will run the Prism algorithm within the SAIL databank to generate risk scores linked to health service usage data for all study patients who do not dissent.

#### Postal questionnaires

We will send postal questionnaires to sampled patients at three points—baseline, 6 and 18 months after Prism implementation in the first study practice. The questionnaire is made up of three validated tools: the adapted Client Service Receipt Inventory, CRSI [31] (to capture individual health service usage data), the Quality of Care Monitor (QCM) [32] and the SF-12 [33] to measure patient outcomes. We will recruit random samples each of 800 patients at each time point to complete the questionnaires (i.e. a minimum of 20 per practice based on 40 participating practices) stratified across the spectrum of risk (see Table 2).

**Table 2:** Details of questionnaire sampling by risk level

**Prism risk level (default score range)**	**Proportion of sample %**	**Sample (number of patients) for screening in each practice**
Level 4 (50 to 100)	20	15
Level 3 (20 to 50)	50	35
Level 2 (10 to 20)	15	10
Level 1 (0 to 10)	15	10
Total sample	100	70

As higher risk patients are likely to receive more intensive resources, we shall oversample at the higher risk levels (3 and 4). Our sampling approach will also take account of an expected reduced response rate from higher risk patients—many of whom will have multiple chronic conditions. Practice patients less than 18 or greater than 100 years of age, recently deceased or moved will be excluded from the sampling frame. Random sampling of the patient population will be carried out on the anonymous Prism data by the Prism data providers (NHS Wales Informatics Service; NWIS). The selected patients will only be identifiable at practice level. Once selected, the GPs from participating practices will assess the suitability of the patients to receive the Prismatic questionnaire by screening the list of sampled patients. Examples of reasons for patient exclusion will include patients that lack capacity, those who do not have support to help them complete the questionnaire and patients who may be caused distress by completing the questionnaire. Questionnaires packs (letter from GP, information sheet, consent form, questionnaire, postage-paid envelope) will be sent directly from participating practices to selected patients. We will gain consent from patients to participate in the trial. Completed questionnaires and consent forms will be returned directly to the study team. Only following consent will the study team gain access to patient demographic information (name, date of birth, address, etc.). The practices will send out a second questionnaire pack to those patients who have not responded to the first if no reply has been received after 2 weeks.

We will adopt the same basic design for each of the two later surveys. Recruited baseline practice patients will be screened again at the later time points by their GPs to ensure that they are still suitable to participate and that none of the participants have died. We will re-sample the GP practice population to replace any losses and to ensure that we have the same number of patients from each practice at these later time points. We will stratify the replacement sample by age, sex and risk stratum to match those removed from the sample.

#### Focus groups and interviews

We will collect qualitative data from GPs and practice staff at baseline and post implementation to explore current practice in chronic conditions management and processes of change initiated by Prism. Questions will address attitudes, expectations and experience relating to predictive risk stratification and specifically the Prism tool, including barriers and facilitators to use.

At baseline, before Prism is activated in the first intervention practices we will conduct four focus groups with staff from general practices, two in one locality where geography suggests a natural division and one each in the other two locality areas. GPs unable to take part in a focus group will be offered an interview by telephone or face-to-face. We will also conduct focus groups with area-wide senior managers and community-based practitioners, one at baseline and one at the end of the intervention period. Focus groups will allow exploration of different views and experiences and encourage group interaction [34].

In order to gain more in-depth information about adoption and use and perceptions of effectiveness, we will undertake one-to-one interviews with staff following Prism implementation. We will purposively sample half the participating practices (20, based on 40 participating practices) and interview Prism user(s) at two time points—3 and 9 months after Prism implementation—face-to-face or by telephone. This will allow us to explore changes in adoption and use over time. We will administer a questionnaire to the other half of participating practices, also at 3 and 9 months but before interviews take place. Questionnaire responses will inform our interviews and enable us to see divergence or concurrence across all participating practices.

We will also interview three senior managers within ABM UHB during implementation to explore area-wide issues related to patient management and the effects of Prism in GP practices. Interviews will allow us to explore in detail respondents’ views about Prism and the use of the tool in their area [35].

In order to gain political, managerial and historical perspectives on the development and implementation of Prism, we will undertake further interviews with managers, policy makers and health services commissioners (*n* = 5) with an all-Wales perspective, face to face or by telephone at baseline. In addition, we will carry out interviews with respondents from non-participating Health Board sites across Wales (*n* = 6) in order to examine their experience of Prism and their perspective on its role and potential.

### Sample size and power

The total of 2,400 respondents will allow us to detect changes between current intervention and control sites in resource use across the spectrum of risk. For example we shall have 80% power when using a 5% significance level to detect changes of 15% in the proportion of patients at a defined risk receiving a specified resource, such as case management or support to quit smoking.

### Analysis

The study will comply with the Statistics Standard Operating Procedure (SOP) of the West Wales Organisation for Rigorous Trials in Health (WWORTH), the clinical trials unit at Swansea University. Primary analysis will be by treatment allocated. The primary outcomes are number of emergency admissions per patient and the time to first event (namely emergency admission). The first of these is a count variable and hence can be modelled using a generalised linear model incorporating an appropriate discrete distribution; the second is a measurement variable, subject to right-censoring, and can be modelled using appropriate survival analysis techniques including Cox’s proportional hazards models. Both methods take account of covariates and explanatory factors (including whether the participant’s practice has yet adopted Prism or not); neither methodology makes any normality assumptions. The list of potential explanatory factors and covariates includes baseline observations, time-varying covariates and days at risk.

The technical performance of the Prism tool will be assessed by analysing the data at baseline and across the control phase. We will plot the proportion of patients who experience hospital admissions against the prospective Prism risk score and calculate sensitivity, specificity, positive and negative predictive values. We will control for any confounding effects of Prism implementation during the analysis period by fitting a binary parameter showing whether practices have adopted Prism yet or not.

The perspective of NHS Wales and personal social services will be adopted for the health economic analysis. The costs of implementing Prism in intervention and control sites will be derived from interviews with GP practice staff and with members of the main trial team (e.g. training resources). The costs associated with resulting changes in care processes will be derived from routine data collected by the Prism system from SAIL and by patient-reported information collected by the CSRI questionnaire. Costs will be presented in a tabular format reporting the estimated resource quantities and unit costs attached. The estimation of the size of the differences (means and standard deviations) in resource use between intervention and control sites within each of the four risk strata and overall will be calculated and valued in monetary terms using published unit costs (with year of reported cost reported) [36]. The primary health economic analysis will be the calculation of the incremental cost per emergency admission avoided in a cost-effectiveness analysis and will produce a tabular representation of costs versus changes in primary and secondary outcomes in a cost-consequences analysis. The incremental cost per quality-adjusted life year (cost/QALY) will be calculated in a cost-utility analysis using SF-6D utility scores derived from SF-12 patient questionnaire data. A series of univariate sensitivity analyses will be carried out to determine the extent to which changes in the basic assumptions of the economic analysis affect the incremental cost-effectiveness ratio.

We will record and transcribe focus groups and interviews and analyse them thematically. This is a systematic and transparent method of analysis that generates themes from the explicit and implicit ideas contained in the original accounts of participants. One researcher will lead the analysis with two others independently supporting key stages of coding, generating themes and interpretation and encouraging a critical stance to test and confirm findings [35,37,38].

### Project management

The trial has been adopted by WWORTH and we will adhere to all relevant WWORTH standard operating procedures (SOPs) in the conduct, management and monitoring of the study. The strategic management of the trial will be the responsibility of a Research Management Group (RMG) meeting quarterly and comprising the Chief Investigator, all co-applicants, all research staff, two service users and two local participating General Practitioners. Operational management will be the responsibility of the Research Team meeting every month and comprising the researchers, clerical support, the Principal Investigator and one of the co-applicants. HAH will be Research Manager responsible for the operational management of the project from day to day. The PI and Research Manager will ensure adherence to the planned timescale and detailed plans for data management and analysis. A data management task and finish group will oversee all data management and analysis issues. The WWORTH SOP on data management will be used to develop a data management plan, outlining details of data entry, coding, security and storage, including any related processes to promote data quality. An independent Trial Steering Committee (TSC) will provide overall supervision for the study and ensure the rigorous conduct of the trial. It will meet twice a year and be made up of an independent chair with an interest in emergency care, an academic in primary care, a consultant in public health, a statistician and two service users (with no previous involvement in the trial). We will adopt the principles outlined in WWORTH’s SOPs on Quality Assurance and independent trial monitoring will be carried out through WWORTH.

### Including service and research users

In accordance with the WWORTH Standard Operating Procedure for Service User Inclusion [39], we have recruited two service users who will actively participate throughout the study as members of the Research Management Group. They were recruited through SUCCESS (Service Users with Chronic Conditions Encouraging Sensible Solutions), a group of patients and carers involved in research linked to the chronic conditions management policy in Wales (http://www.invo.org.uk/posttypeconference/an-alternative-success-model/) The two service user representatives contribute views from the wider SUCCESS group.

### Ethics and dissemination

The Multi-Centre Research Ethics (MREC) Committee for Wales has given full ethical approval for the study (reference 10/MRE09/25). R&D permissions have been granted across Wales. We have received Information Governance Review Panel (IGRP) permission for use of the SAIL databank. We will seek further approval for any proposed changes to the trial design or conduct with the MREC and relevant R&D committees via amendment reports.

We will comply with the CONSORT guidelines [40]. We will present study results at national and international conferences and publish them in peer-reviewed and clinical journals. We have produced a publication plan and authorship agreement for dissemination of the study findings. Only those individuals who fulfil the authorship criteria will be included as authors on final publications.

## Discussion

There is a lack of evidence regarding how well predictive risk tools work in supporting the management of patients. The proposed study will provide information on costs and effects of Prism; how it is used in practice, barriers and facilitators to its implementation; and its perceived value in supporting the management of patients with and at risk of developing chronic conditions. These findings will have UK and international relevance at a time of heightened focus on chronic conditions management and predictive modelling.

## Trial status (August 2013)

The PRISMATIC trial is currently underway and we have now recruited 32 General Practices across the AMB UHB area to take part. Baseline qualitative data collection (staff focus groups and interviews) were carried out between October 2012 and January 2013. Baseline patient questionnaire distribution began in April 2013.

### Consent

Written informed consent was obtained from the patient for the publication of this report and any accompanying images.

## Abbreviations

ABM UHB: Abertawe bro morgannwg university health board; CSQI: Client service receipt inventory; EDDS: Emergency department data set; IGRP: Information governance review panel; NHS: National health service; NWIS: NHS wales informatics service; PEDW: Patient episode database for wales; QALY: Quality-adjusted life year; QCM: Quality of care monitor; SAIL: Secure anonymised information linkage; SOP: Standard operating procedure; WWORTH: West Wales organisation for rigorous trials in health; WAO: Wales audit office.

## Competing interests

The authors declare that they have no competing interests.

## Authors’ contributions

HAH, HAS, ITR, BAE, AP, PH, DW, LL and CJP were responsible for formulating the overall research question and the design of the study. MRK provided input to revised versions of the protocol. BAE and AP were responsible for designing the qualitative aspects of the study and BS, CJP and DF for the health economics components. AW was responsible for leading the data analysis and statistical plan for the study. DW and LL provided input regarding the Prism tool, IT aspects and service delivery. PH provided expertise regarding social care. JH provided general practice input and was involved in developing the intervention and setting up the study within ABMU. HAH wrote the first draft of this manuscript and was responsible for the revisions. All authors provided input into the drafting of the manuscript and read and approved the final version.
